# The association between plate location and hardware removal following ulna shortening osteotomy: a cohort study

**DOI:** 10.1177/17531934221089228

**Published:** 2022-04-11

**Authors:** Joris S. Teunissen, Sanharib Al Shaer, Brigitte P.A. van der Heijden, Ruud W. Selles, Steven E.R. Hovius, Oliver T. Zöphel, on behalf of the Hand Wrist Study Group

**Affiliations:** 1 Department of Plastic, Reconstructive and Hand Surgery, Radboud Institute for Health Sciences, Radboudumc, Nijmegen, The Netherlands; 2 Hand and Wrist Center, Xpert Clinics, Amsterdam, The Netherlands; 3 Department of Surgery, Medisch Spectrum Twente, Enschede, The Netherlands; 4 Department of Plastic, Reconstructive and Hand Surgery, Jeroen Bosch Ziekenhuis,’s-Hertogenbosch, Brabant, The Netherlands; 5 Department of Plastic, Reconstructive and Hand Surgery, Erasmus MC, Rotterdam, The Netherlands; 6 Department of Plastic, Reconstructive and Hand Surgery, Ziekenhuisgroep Twente, Almelo, The Netherlands

**Keywords:** Complication, hardware removal, ulna shortening osteotomy, wrist

## Abstract

Hardware removal after ulna shortening osteotomy is common. We evaluated the association between plate location and hardware removal rate in 326 procedures in 321 patients with a median follow-up of 4.3 years (IQR 3.3) and corrected for confounding variables and did survival analyses. Complications were scored using the International Consortium for Health Outcome Measurement complications in Hand and Wrist Conditions tool. The 1-year and 5-year reoperation rates for hardware removal were 21% and 46% in the anterior group versus 37% and 64% in the dorsal group. Anterior plate placement was independently associated with a decreased immediate risk of hardware removal. Higher age, male sex and treatment on the dominant side were also associated with a reduced risk of hardware removal. We did not find a difference in hardware removal rates between freehand or jig-guided ulna shortening osteotomies. We noted perioperative problems in 3% of the procedures and complications in 20%.

**Level of evidence:** III

## Introduction

Ulnar shortening osteotomy (USO) is frequently performed for various ulnar-sided wrist disorders, such as ulnar impaction syndrome, irreparable degenerative triangular fibrocartilage complex tears and mild distal radioulnar joint (DRUJ) instability (Chun and Palmer, 1993; Iwatsuki et al., 2014; Loh et al., 1999; Tatebe et al., 2005).

Despite good outcomes (Chun and Palmer, 1993; Iwatsuki et al., 2014; Stockton et al., 2015; Tatebe et al., 2005), previous studies have reported high reoperation rates after USO. Plate removal due to hardware irritation seems to be the most prevalent cause for reintervention (Chan et al., 2016; Verhiel et al., 2020). The rate of hardware removal varies largely (0–71%) between studies (Fricker et al., 1996; Kitzinger et al., 2007). Hardware removal is not without risk, as refractures and other complications may occur (Vos et al., 2012; Yao et al., 2014).

There is ongoing debate about optimal plate location to decrease plate irritation and the need for removal (Das De et al., 2015; Kitzinger et al., 2007; Megerle et al., 2015; Pomerance, 2005). Some authors advocate anterior placement of the plate (Chen and Wolfe, 2003; Kitzinger et al., 2007) or dorsal placement (Das De et al., 2015), while others did not find a significant difference in complication rates between plate locations (Megerle et al., 2015; Verhiel et al., 2020).

Few retrospective studies have reported on predictors for hardware removal (Chan et al., 2016; Das De et al., 2015; Jungwirth-Weinberger et al., 2016; Pomerance, 2005; Verhiel et al., 2020). Factors other than plate location that are associated with an increased rate of hardware removal include heavy physical work (Labosky et al., 1990) and older age (Verhiel et al., 2020).

This study investigates whether the position of the fixation plate on the ulna influences the immediate risk of hardware removal after USO when adjusting for potential confounding variables, and what other factors are associated with an increased rate of hardware removal. Additionally, we report the peri- and postoperative complications associated with hardware removal.

## Methods

In this multicentre retrospective cohort, we studied patients who underwent USO between July 2011 and November 2019 at Xpert Clinics, the Netherlands. Our institution grew from one clinic with two hand surgeons to 18 clinics with 23 hand surgeons and over 150 hand therapists during the study period. Our study was conducted according to guidelines from the ‘Strengthening the Reporting of Observational Studies in Epidemiology’ statement (Vandenbroucke et al., 2007). The local medical research ethical committee of the Erasmus University Medical Centre approved the study. All patients provided written consent.

### Participants

The patients included in this study were part of the Hand and Wrist Cohort, a routine measurement system for quality registration purposes (Selles et al., 2020). We identified all patients with a treatment code of USO between 2011 and 2019, and the first authors (JST and SAS) manually checked these entries within the patient charts to avoid misclassification (e.g. when surgery was cancelled, or another procedure was performed). Bilateral procedures were included in the study since they do not introduce significant dependency problems in register studies (Ranstam et al., 2011). We excluded patients when the plate position or plate type could not be retrieved from their charts or radiographs or when treatment codes were indexed wrongly in the database.

### Variables and measurements

Age, sex, type of work, symptom duration, treatment side and hand dominance were routinely registered by a certified hand therapist during admission. In addition, other patient characteristics, such as smoking status at the time of treatment (yes/no), weight and height, were self-reported by web-based secure questionnaires (GemsTracker©, Rotterdam, The Netherlands).

Electronic patient files and radiographs were evaluated for operative variables by the authors (JST, SAS, EPAvdH and OTZ). Surgery was performed by 19 Federation of European Societies for Surgery of the Hand (FESSH) certified hand surgeons with experience levels 3 (*n* = 8), 4 (*n* = 9) and 5 (*n* = 2) (Tang and Giddins, 2016). All USOs were performed at the level of the distal diaphysis using a diagonal cut. Based on preoperative ulnar variance, the median amount of shortening was 4 mm (IQR 1). The total number of annual USOs increased over time due to clinic growth (Online Figure S1) (Selles et al., 2020). While both plate locations were used during the entire study period, we observed a decrease in dorsal placement and an increase in anterior placement since 2017. In earlier years, a freehand technique was mostly used (AO, Davos, Switzerland), whereas this was gradually replaced by jig-guided osteotomies (Acumed®, Hillsboro, OR, USA; Recos® KLS Martin, Tuttlingen, Germany; Trimed®, Santa Clarita, CA, USA; Medartis®, Basel, Switzerland). Generally, the fixation plates were placed 3 cm proximal to the ulnar head on the anterior or dorsal surface of the ulna.

The primary outcome was the rate of hardware removal, which is not routinely performed in the Netherlands, but may be indicated on clinician-based arguments or patient-based symptoms (Vos et al., 2012). Patient-based symptoms are considered a valid reason for hardware removal (Vos and Verhofstad, 2013). We only considered hardware removal after careful clinical and radiographical affirmation of bone union and informed consent after shared decision making. The indication for hardware removal was subtracted from the patient records and classified, according to a review from Vos and Verhofstad (2013), as (1) surgeon derived arguments (such as broken material, infection or tendon rupture); (2) patient’s requests (such as: ‘it does not belong to my body’ and litigations); (3) patient’s complaints (such as pain, swelling, paraesthesia, problems with daily living or cosmetic issues due to plate prominence).

Perioperative findings and complications after hardware removal were subtracted from the electronic patient files and scored following the International Consortium for Health Outcome Measurement Complications in Hand and Wrist Conditions (ICHAW) (Wouters et al., 2021). This tool classifies surgical complications into different grades (I-III; a higher grade is more severe) based on the treatment required (Online Table S1).

### Statistical methods

Time-to-event (hardware removal) was calculated in weeks. In patients who did not undergo hardware removal, we calculated event-free time by subtracting the date of USO and the last evaluation of their patient record (minimal 1.5 years after initial USO). Patients who did not undergo hardware removal during the study period were censored after their recorded event-free time had surpassed to account for variations in follow-up time and minimize bias (Kirch, 2008). Kaplan–Meier survival analyses were performed to evaluate the cumulative incidence of hardware removal, including 95% confidence intervals (CI) at 1, 2 and 5 years after initial USO. Differences between groups were tested using a log-rank test. The weeks in which participants were censored are marked with a ‘+’ in the Kaplan–Meier curve.

We used a Cox proportional hazards model to estimate adjusted hazard ratios (HRs) of hardware removal with 95% CI for each variable in the model. The following variables were included in the model: sex, age, body mass index (BMI), smoking, type of work, treatment side, plate location, surgeon expertise level and plate type. Plate type was used instead of osteotomy technique (freehand versus jig) since plates from different manufacturers have distinct profiles. A HR larger than one was interpreted as an increased hazard of hardware removal, and an HR smaller than one as a decreased hardware removal hazard (Brody, 2016). The hazard is the immediate risk of experiencing an event at time *t* (Sashegyi and Ferry, 2017). We tested the proportional hazards assumption using the Schoenfeld residuals.

The number of patients treated during the study period determined the sample size. Sample size calculations for Cox models primarily depend on simulation studies (Scosyrev and Glimm, 2019). We adhered to the recommended minimum of ten events per variable (Concato et al., 1995; Peduzzi et al., 1995).

To investigate whether a difference in hardware removal rates could be explained by healthcare-avoiding behaviour during the COVID-19 lockdown, we conducted a sensitivity analysis by only including patients treated before March 2018, which was 2 years before the lockdown (Government of the Netherlands, 2021). For all analyses, a *p*-value <0.05 was considered statistically significant.

## Results

We identified 351 USO records in the database and excluded 25 wrongly indexed ones (e.g. the patient underwent a treatment other than USO). The study population included 326 procedures (performed in 321 patients). Patient characteristics are displayed in Table 1. The median patient age was 46 (IQR 22.8) and 67% were female. The median time between USO and last electronic patient files check was 4.3 years (IQR 3.3).

**Table 1.** Characteristics of the 326 procedures (321 patients).

Variable	All (*n* = 326)	Dorsal (*n* = 199)	Anterior (*n* = 127)
Age [years], median (IQR)	46 (23)	44 (22)	50 (21)
Female patients, *n* (%)	219 (67)	130 (65)	89 (70)
BMI, median (IQR)	26 (4)	26 (4)	26 (5)
Smoker, *n* (%)	81 (25)	43 (22)	38 (30)
Treatment side, *n* (%)			
Dominant, non-dominant	183 (56)	107 (54)	76 (60)
Non-dominant	143 (44)	92 (46)	51 (40)
Type of work, *n* (%)			
None	93 (28)	53 (27)	40 (31)
Light	67 (21)	42 (21)	25 (20)
Moderate	102 (31)	62 (31)	40 (31)
Heavy	64 (19)	42 (21)	22 (17)
Plate type, *n* (%)			
AO	113 (35)	85 (43)	28 (22)
Acumed	200 (61)	111 (56)	89 (70)
Medartis	3 (1)	2 (1)	1 (1)
KLS Martin	7 (2)	0 (0)	7 (6)
Trimed	3 (1)	1 (1)	2 (2)

IQR: interquartile range; BMI: body mass index; *n*: number; AO: Arbeitsgemeinschaft für Osteosynthesefragen.

USO plate was removed in 181 patients. In 179 (99%), the indication for hardware removal was based on patient complaints (painful/irritating hardware *n* = 174; wrist motion limitation *n* = 34; paraesthesias *n* = 6; cold intolerance *n* = 1). In two patients, the decision was not based on complaints: one patient had radiological bone atrophy of the ulna, and the other was less than 18 years old and was beginning a professional sports career.

**Figure 1.**
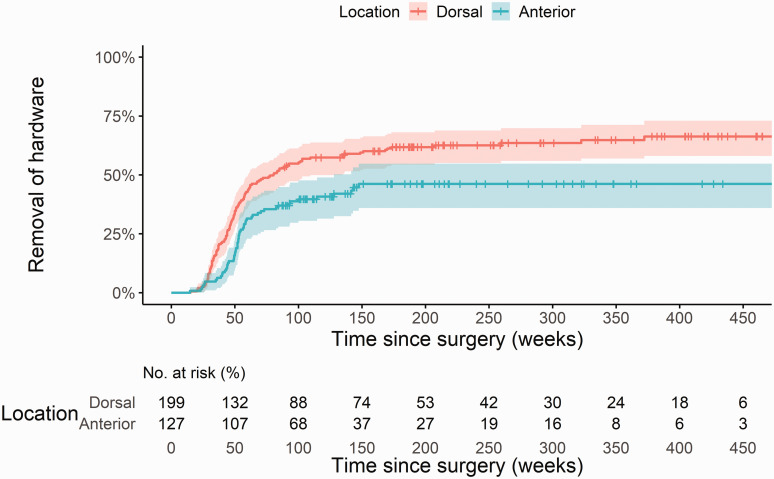
Kaplan–Meier curve including 95% for hardware removal after ulna shortening osteotomy based on plate location (anterior or dorsal). The number of patients at risk in each group is shown for every 50 weeks since USO.

The timing of hardware removal varied from 15 to 372 weeks after USO, and 80% were performed between 29 and 103 weeks (Online Figure S2). The Kaplan–Meier curves stratified for plate location are as shown in Figure 1. After 5 years, the cumulative hardware removal rate was 64% (CI 56 to 70%) in the dorsal group and 46% (CI 36 to 55%) in the anterior group (*p* = 0.001). The hardware removal rate was also lower in the anterior group in the sensitivity analysis (*p* = 0.034) and when excluding the Recos, Trimed and Medartis plates (*p* < 0.001). We found no difference based on the osteotomy technique (*p* = 0.47; Online Figure S3). Event rates at other time points are shown in Online Table S2. The median time until hardware removal was 80 weeks in the dorsal group, meaning that at 80 weeks after the USO, 50% of the plates had been removed. The median time in the anterior group could not be calculated as only 46% of the plates had been removed by the end of the study period.

### Factors associated with hardware removal

The rate of hardware removal was lower in the anterior placement group with an adjusted HR of 0.62 (CI 0.44 to 0.89; *p* = 0.008) (Online Table S3). This means that having an anterior fixation plate was associated with a 38% reduced hazard of hardware removal compared with dorsal fixation when correcting for confounding variables. Older age (HR 0.88; CI 0.78 to 0.97; *p* = 0.015) was independently associated with a reduced hazard of hardware removal (12%/10 years) and male sex with a 32% reduced hazard compared with females (HR 0.68, CI 0.48 to 0.96; *p* = 0.029). Treatment on the non-dominant side was associated with a 37% increased hazard of hardware removal compared with treatment on the dominant side (HR 1.37, CI 1.01 to 1.83; *p* = 0.038).

### Perioperative findings and complications

Perioperative problems were noted in six patients (3%) and complications in 37 patients (20%) (Table 2). Twenty (11%) had a Grade I, 11 (6%) Grade II, four (2%) Grade IIIA and three (1%) patients had a Grade IIIB complication. Based on plate location, we did not observe a difference in perioperative problems (*p* = 0.54) and complications (*p* = 0.48). Three patients (2%) had a refracture of the ulna after hardware removal; the time between USO and hardware removal in these patients was 31, 44 and 58 weeks (Table 3).

**Table 2.** Problems during hardware removal (181 procedures) after ulna shortening osteotomy and complications following ICHAW (stratified based on plate location (Dorsal *n* = 126; Anterior *n* = 55)).

	Overall	Dorsal	Anterior	*p*-value^a^
*Perioperative problems*				
None	175 (97%)	123 (98%)	52 (95%)	0.541
Difficulties with removal due to bone overgrowth	1	0	1	
Overturned screw	1	1	0	
Failing nerve block	1	1	0	
Ulnar nerve in scar tissue, neurolysis performed to get to the plate	1	0	1	
Larger incision is needed to remove the plate	2	1	1	
*Complications*				
None	144 (80%)	98 (78%)	46 (84%)	0.484
*Grade I*				
None	161 (89%)	110 (87%)	51 (93%)	0.416
(Unspecified) pain:				
Hand therapy and splint	5	5	0	
Analgesics	1	1	0	
Acute postoperative pain: Analgesics	3	1	2	
EPL dysfunction (related to anaesthesia): hand therapy and splint	1	1	0	
Ulnar nerve sensibility disturbances including numbness:				
De-sensibilization therapy	1	0	1	
Expectative	3	2	1	
Scar tenderness:				
Scar massage therapy	1	1	0	
Expectative	4	4	0	
Swelling: coban glove	1	1	0	
*Grade II*				
None	170 (94%)	118 (94%)	52 (95%)	0.999
TVS: corticosteroid injection	1	1	0	
Pain: corticosteroid injection	4	3	1	
Wound infection: antibiotics	1	1	0	
Postoperative bleeding: bandages	2	1	1	
Hematoma: analgesic	1	0	1	
Refracture: cast	2	2	0	
*Grade III*				
None	174 (97%)	121 (96%)	53 (96%)	0.999
*A*				
Abscess: drainage	2	1	1	
Skin irritation: stitch removal	2	1	1	
*B*				
Hematoma: drainage	1	1	0	
Postoperative bleeding: Exploration and coagulation.	1	1	0	
Refracture: refixation with plate	1	1	0	

a The *p*-value was calculated between the volar and dorsal group, using a chi-squared test.

ELP: extensor pollicis longus; TVS: tendovaginitis stenosans; *n*: number.

**Table 3.** Characteristics of the three patients with a refracture after hardware removal.

Variable	Patient 1	Patient 2	Patient 3
Age	19 years	17 years	33 years
Sex	Male	Female	Male
BMI	25	25	26
Smoking status	Yes	No	No
Treatment side	Dominant	Non-dominant	Dominant
Type of work	None	None	Light
Plate position	Dorsal	Dorsal	Dorsal
Plate	Acumed	Acumed	AO
Removal after USO	44 weeks	31 weeks	58 weeks
Mechanism details	Unknown	Traffic accident	Heavy load-lifting

BMI: body mass index; AO: Arbeitsgemeinschaft für Osteosynthesefragen; USO: ulna shortening osteotomy.

## Discussion

An explanation for the difference in hardware removal rates based on plate location may be the anatomical advantage of anterior placement with thicker soft tissue coverage over the hardware (Pomerance, 2005). Also, the extensor carpi ulnaris may be prone to subluxing over a dorsal plate, whereas this is unlikely for the flexor carpi ulnaris over an anterior plate.

Several studies have compared the rate of hardware removal for different plate locations and found contradictory results. Das De et al. (2015) found significantly lower reoperations in the dorsal group (1/16; 6%) compared with the anterior group (6/18; 50%). Three other studies (*n* = 35 to 98) found no statistical differences based on plate location (Fufa et al., 2014; Megerle et al., 2015; Verhiel et al., 2020). However, the results of previous studies should be interpreted with caution as they may have been underpowered to detect a statistical difference and did not adjust for potential confounders. Also, the follow-up duration should be considered when reporting the rate of hardware removal, as some patients opt for hardware removal even after more than 4 years of follow-up.

We did not find a difference in hardware removal rates based on different types of fixation plates, which is in line with the results of Verhiel et al. (2020). Jungwirth-Weinberger et al. (2016) showed that using the new locking 2.7 mm compression plate did not decrease the number of hardware removals due to hardware irritation and concluded that plate location is more important than its thickness, size or design.

Besides plate location, we identified some sociodemographic factors independently associated with hardware removal. First, younger age was associated with higher rates of hardware removal. The immediate risk of hardware removal decreased by 12% for every 10 years in age. A possible explanation is that younger patients have a more active lifestyle and experience more discomfort from the friction of the plate. A previous study also advocated plate removal in younger patients after bone union because of the prolonged exposure to metal corrosion and metal ions (Labosky et al., 1990). However, this should no longer be a relevant consideration with the newer alloys (Vos and Verhofstad, 2013). Also, surgeons might have had a lower threshold to remove the plate in younger patients; for example, one surgeon in our study recommended removing the plate in one asymptomatic patient younger than 18 years in anticipation of future sports-related future injuries. Second, female patients had a 32% increased risk of hardware removal as compared with males. A possible cause for the higher incidence of hardware removal in women is that they experience more complaints from the hardware due to less robust soft tissue cover. Third, USO performed on the non-dominant side was associated with an increased instantaneous risk of 37% as compared with the dominant side. Some patient dossiers mention plate irritation when wearing watches or jewellery, which might be an explanation. We expected the BMI and the physical level of work to influence the reoperation rate; however, these factors were not found to be significant. Verhiel et al. (2020) also investigated hardware removal rates (98 patients) for various sociodemographic variables using bivariate analyses. In line with our findings, they found that patients undergoing hardware removal were younger but there were no differences according to the BMI or type of work. In addition, they did not report any differences based on sex and treatment side.

As the newly developed ICHAW classification was used in this study, comparisons with other studies should be made with caution as their complication scoring protocol may not be comparable with ICHAW. In our study, the bleeding rate was 3%, infection rate was 1% and refracture rate was 2%. These rates do not differ from other commonly performed hand and wrist surgeries. Two of the three patients who had a refracture had their plate removed in the first year after USO. While 94 of the 96 plates that were removed in the first year after USO did not lead to refracture, early removal should be performed with caution. While a previous study reported that union is achieved after a mean of 4 to 5 months after USO, complete consolidation was only seen on radiographs after 16 to 20 months (Kang et al., 2021). Therefore, the ulna may be still at greater risk of refracture in the face of a new injury.

This study has a few limitations. First, some patients could have had their hardware removal elsewhere, leading to an underestimation of the true incidence. We considered using the last clinical note at the end of the follow-up, however, this would have resulted in selection bias as patients that returned to the clinic for hardware removal or other hand and wrist complaints were followed longer, whereas satisfied patients would have been excluded. Furthermore, we assumed that the plate locations were equally distributed in patients that underwent hardware removal elsewhere, thereby not affecting the HR. Second, there were no strict predefined indications justifying hardware removal. Third, the incidence of symptoms, such as wrist motion impairment, paraesthesia and cold tolerance, should be interpreted with caution as they are likely underestimated due to underreporting in the patients’ charts.

Future prospective studies could incorporate additional measurements (such as dynamic ultrasound) before hardware removal to investigate if patients’ complaints relate to objective clinical signs. Furthermore, the role of psychosocial aspects, such as pain catastrophizing, mental distress and illness perception on hardware irritation, should be investigated, as these are known to influence the outcome in other types of musculoskeletal surgery.

## Supplemental Material

sj-pdf-1-jhs-10.1177_17531934221089228 - Supplemental material for The association between plate location and hardware removal following ulna shortening osteotomy: a cohort study Click here for additional data file. Supplemental material, sj-pdf-1-jhs-10.1177_17531934221089228 for The association between plate location and hardware removal following ulna shortening osteotomy: a cohort study by Joris S. Teunissen, Sanharib Al Shaer, Brigitte P.A. van der Heijden, Ruud W. Selles, Steven E.R. Hovius, Oliver T. Zöphel and on behalf of the Hand Wrist Study Group in Journal of Hand Surgery (European Volume)

sj-jpg-2-jhs-10.1177_17531934221089228 - Supplemental material for The association between plate location and hardware removal following ulna shortening osteotomy: a cohort study Click here for additional data file. Supplemental material, sj-jpg-2-jhs-10.1177_17531934221089228 for The association between plate location and hardware removal following ulna shortening osteotomy: a cohort study by Joris S. Teunissen, Sanharib Al Shaer, Brigitte P.A. van der Heijden, Ruud W. Selles, Steven E.R. Hovius, Oliver T. Zöphel and on behalf of the Hand Wrist Study Group in Journal of Hand Surgery (European Volume)

sj-pdf-3-jhs-10.1177_17531934221089228 - Supplemental material for The association between plate location and hardware removal following ulna shortening osteotomy: a cohort study Click here for additional data file. Supplemental material, sj-pdf-3-jhs-10.1177_17531934221089228 for The association between plate location and hardware removal following ulna shortening osteotomy: a cohort study by Joris S. Teunissen, Sanharib Al Shaer, Brigitte P.A. van der Heijden, Ruud W. Selles, Steven E.R. Hovius, Oliver T. Zöphel and on behalf of the Hand Wrist Study Group in Journal of Hand Surgery (European Volume)

sj-pdf-4-jhs-10.1177_17531934221089228 - Supplemental material for The association between plate location and hardware removal following ulna shortening osteotomy: a cohort study Click here for additional data file. Supplemental material, sj-pdf-4-jhs-10.1177_17531934221089228 for The association between plate location and hardware removal following ulna shortening osteotomy: a cohort study by Joris S. Teunissen, Sanharib Al Shaer, Brigitte P.A. van der Heijden, Ruud W. Selles, Steven E.R. Hovius, Oliver T. Zöphel and on behalf of the Hand Wrist Study Group in Journal of Hand Surgery (European Volume)

sj-jpg-5-jhs-10.1177_17531934221089228 - Supplemental material for The association between plate location and hardware removal following ulna shortening osteotomy: a cohort study Click here for additional data file. Supplemental material, sj-jpg-5-jhs-10.1177_17531934221089228 for The association between plate location and hardware removal following ulna shortening osteotomy: a cohort study by Joris S. Teunissen, Sanharib Al Shaer, Brigitte P.A. van der Heijden, Ruud W. Selles, Steven E.R. Hovius, Oliver T. Zöphel and on behalf of the Hand Wrist Study Group in Journal of Hand Surgery (European Volume)

sj-jpg-6-jhs-10.1177_17531934221089228 - Supplemental material for The association between plate location and hardware removal following ulna shortening osteotomy: a cohort study Click here for additional data file. Supplemental material, sj-jpg-6-jhs-10.1177_17531934221089228 for The association between plate location and hardware removal following ulna shortening osteotomy: a cohort study by Joris S. Teunissen, Sanharib Al Shaer, Brigitte P.A. van der Heijden, Ruud W. Selles, Steven E.R. Hovius, Oliver T. Zöphel and on behalf of the Hand Wrist Study Group in Journal of Hand Surgery (European Volume)
