# Supplementary material for: The association between plate location and hardware removal following ulna shortening osteotomy: a cohort study
Source: J Hand Surg Eur Vol. 2022 Apr 11;47(8):831–8. doi: 10.1177/17531934221089228 (PMC9459407; doi:10.1177/17531934221089228)
Supplement: sj-pdf-1-jhs-10.1177_17531934221089228 - Supplemental material for The association between plate location and hardware removal following ulna shortening osteotomy: a cohort study [file sj-pdf-1-jhs-10.1177_17531934221089228.pdf]

Online Table S3: Results of the multivariable Cox regression analysis.

Variable	Hazard Ratio	95%CI	*p*-value
Age (each 10 years)	0,88	[0,78 to 0,97]	**0,015**
Sex			
Females	Ref	Ref	
Males	0,68	[0,48 to 0,96]	**0,029**
BMI	1,02	[0,98 to 1,05]	0,424
Smoking			
Yes	Ref	Ref	
No	0,88	[0,62 to 1,25]	0,486
Type of work			
None	Ref	Ref	
Light	1,27	[0,8 to 2,01]	0,303
Moderate	1,3	[0,87 to 1,94]	0,196
Heavy	1,44	[0,89 to 2,31]	0,136
Treatment side			
Dominant	Ref	Ref	
Non-dominant	1,37	[1,01 to 1,83]	**0,038**
Duration of complaints (months)	1,00	[0,99 to 1]	0,614
Plate			
AO	Ref	Ref	
Acumed	1,25	[0,88 to 1,76]	0,213
KLS Martin	1,56	[0,45 to 5,36]	0,483
Medartis	1,58	[0,37 to 6,71]	0,537
Trimed	1,1	[0,25 to 4,84]	0,897

Expertise level[a]			
III	Ref	Ref	
IV	1,47	[0,89 to 2,43]	0,135
V	1,45	[0,76 to 2,79]	0,261
Location			
Dorsal	Ref	Ref	
Anterior	0,62	[0,44 to 0,89]	**0,008**

CI: Confidence Interval; BMI: Body Mass Index; Ref: reference

[a]According to Tang and Giddins (2016)
