# Supplementary figures and images for: The association between plate location and hardware removal following ulna shortening osteotomy: a cohort study
Source: J Hand Surg Eur Vol. 2022 Apr 11;47(8):831–8. doi: 10.1177/17531934221089228 (PMC9459407; doi:10.1177/17531934221089228)

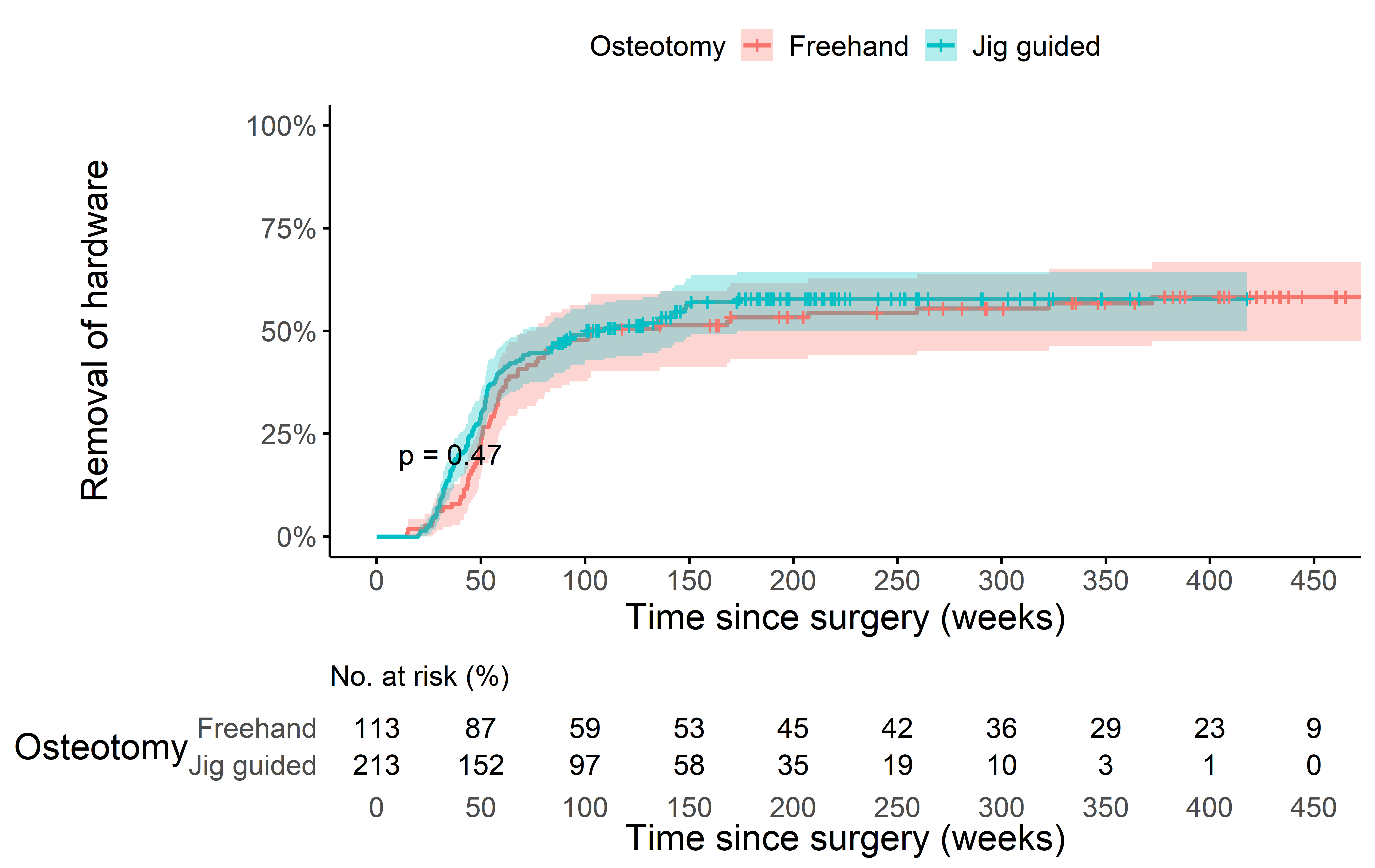

Supplement: sj-jpg-2-jhs-10.1177_17531934221089228 - Supplemental material for The association between plate location and hardware removal following ulna shortening osteotomy: a cohort study [file sj-jpg-2-jhs-10.1177_17531934221089228.jpg]

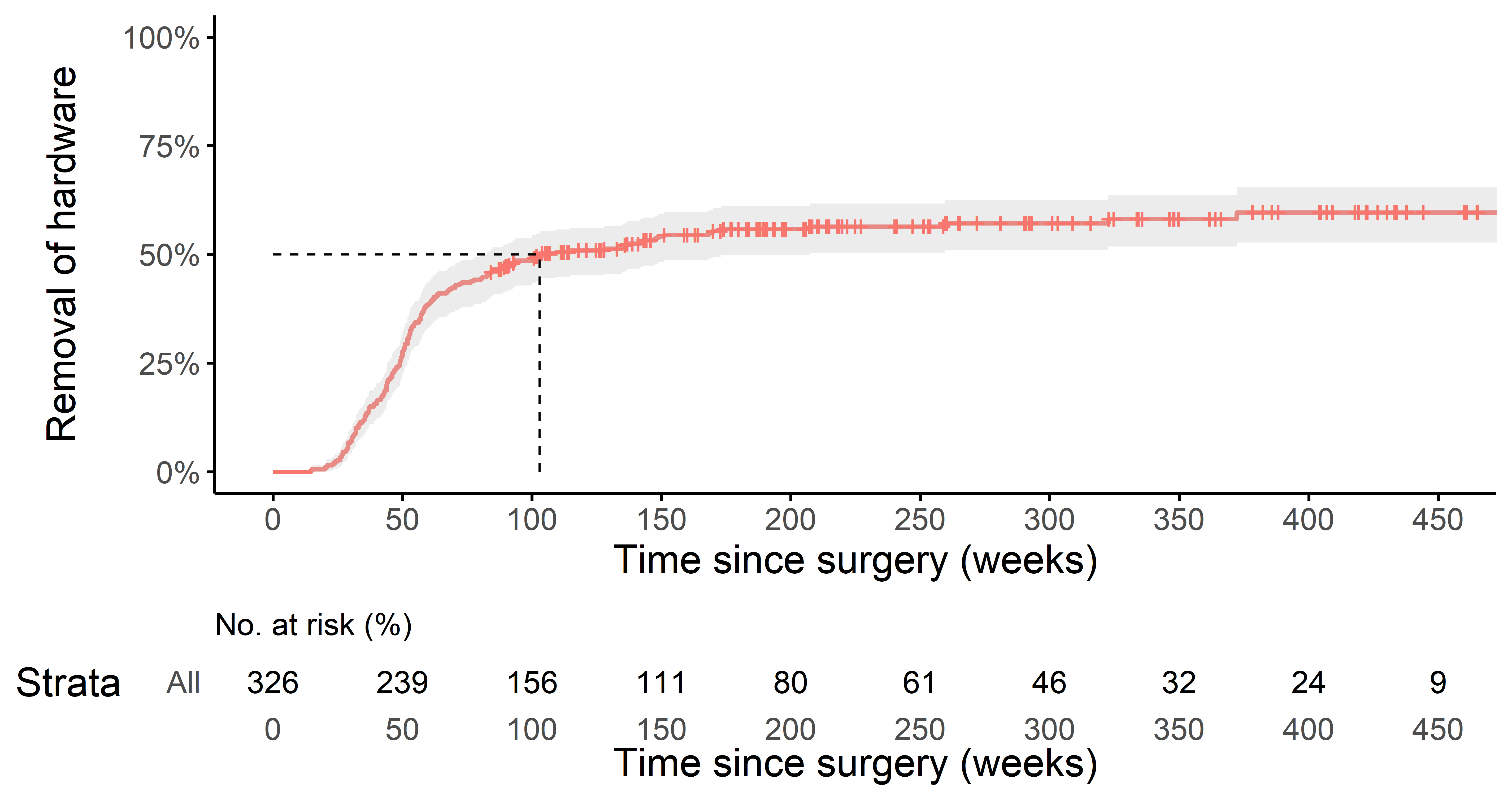

Supplement: sj-jpg-5-jhs-10.1177_17531934221089228 - Supplemental material for The association between plate location and hardware removal following ulna shortening osteotomy: a cohort study [file sj-jpg-5-jhs-10.1177_17531934221089228.jpg]

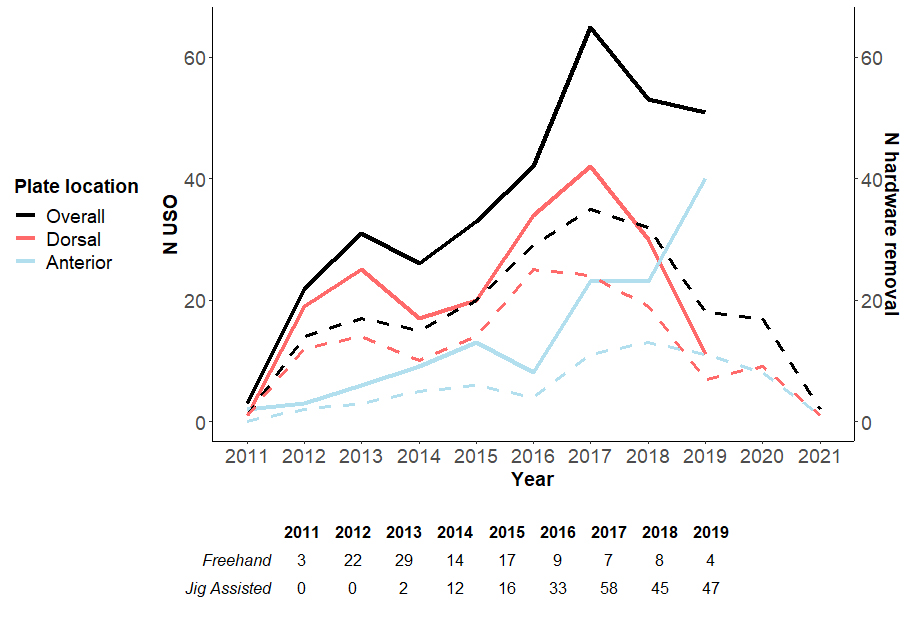

Supplement: sj-jpg-6-jhs-10.1177_17531934221089228 - Supplemental material for The association between plate location and hardware removal following ulna shortening osteotomy: a cohort study [file sj-jpg-6-jhs-10.1177_17531934221089228.jpg]
