# Supplementary material for: The association between plate location and hardware removal following ulna shortening osteotomy: a cohort study
Source: J Hand Surg Eur Vol. 2022 Apr 11;47(8):831–8. doi: 10.1177/17531934221089228 (PMC9459407; doi:10.1177/17531934221089228)
Supplement: sj-pdf-3-jhs-10.1177_17531934221089228 - Supplemental material for The association between plate location and hardware removal following ulna shortening osteotomy: a cohort study [file sj-pdf-3-jhs-10.1177_17531934221089228.pdf]

Online Table 2S: Descriptive results of the survival analyses for all procedures and stratified by plate location.

Variable	Overall (n= 326)	Dorsal n = 199)	Anterior (n = 127)
Number of events	181	126	55
Cumulative event rate			
1-year (CI)	31% (25 to 36%)	37% (30 to 43%)	21% (14 to 28%)
2-years (CI)	50% (44 to 55%)	57% (49 to 63%)	40% (30 to 48%)
5-years (CI)	57% (51 to 63%)	64% (56 to 70%)	46% (36 to 55%)

CI = 95% Confidence interval.
