# Supplementary material for: The association between plate location and hardware removal following ulna shortening osteotomy: a cohort study
Source: J Hand Surg Eur Vol. 2022 Apr 11;47(8):831–8. doi: 10.1177/17531934221089228 (PMC9459407; doi:10.1177/17531934221089228)
Supplement: sj-pdf-4-jhs-10.1177_17531934221089228 - Supplemental material for The association between plate location and hardware removal following ulna shortening osteotomy: a cohort study [file sj-pdf-4-jhs-10.1177_17531934221089228.pdf]

Online Table S1: International Consortium for Health Outcomes Measurement classification of Complications in Hand and Wrist conditions.

Grade	Definition, to occur within the final time point of the relevant track
Grade I:	Any deviation from the normal treatment course without the need for surgical, endoscopic, and radiological interventions. Acceptable therapeutic regimens are extra analgesics and additional hand therapy/ splinting/ cast. This grade includes e.g.: tendinitis, scar tenderness, temporary sensory disturbances, etc. Complex Regional Pain Syndrome is excluded from this grade (see Grade III-C).
Grade II:	Any deviation from the normal treatment course requiring antibiotics, steroid injections, or other pharmacological treatment not listed in Grade I. Also included are wound infections and hematoma's not needing anesthesia. Complex Regional Pain Syndrome is excluded from this grade (see Grade III-C).
Grade III:	Any deviation from the normal treatment course requiring surgical, endoscopic, or radiological intervention. Also, this includes tendinitis, scar tenderness, persistent pain, etc. not responding to conservative therapy, drugs, or injections.
A:	Minor surgical intervention under local anesthesia (e.g., irritating K wire, suture removal subcutaneously)
B:	Major surgical intervention under regional or general anesthesia (e.g., repeat surgery, tenolysis, neurolysis, nerve repair or surgery for tendon rupture, breaking of the plate, non-union, initial prosthesis failure)
C:	Complex Regional Pain Syndrome, diagnosed using Budapest criteria, independent of the initiated treatment
